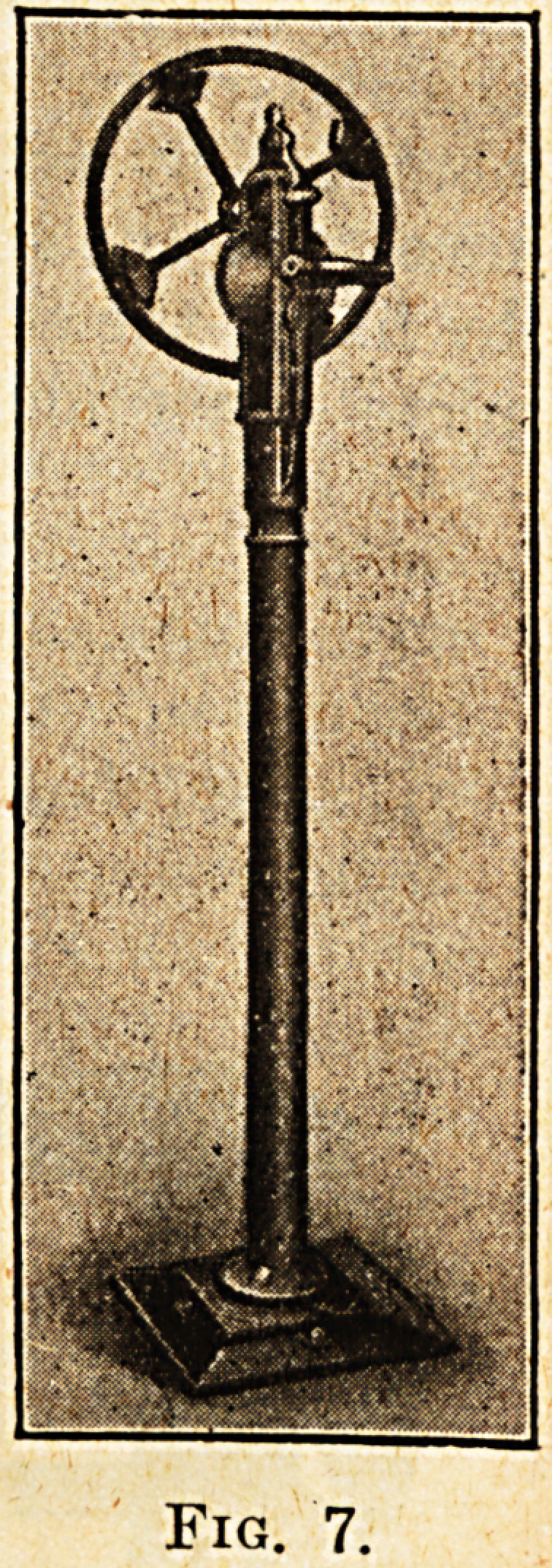# Some Zander Machines Described and Illustrated

**Published:** 1916-11-25

**Authors:** 


### Some Zander Machines Described and Illustrated.

### **Arm Circumduction.**—Fig. 1.

The patient sits on the stool with the arm to be exercised resting in the support A. He then grasps the hand-piece B, which moves up and down the rod C, D. The range of movement required is obtained by adjusting the end of the rod C, D by means of the screw E. Less movement is obtained by fixing the screw E nearer the pivot F, while complete circumduction is obtained by adjusting the rod to the farthest point of the lever at G. If the shoulder joint is not entirely helpless the patient is directed to make as much movement as he is capable of, and the lever and weight thus set in motion continues the movement, carrying the arm gently round in a circle, the hand-piece B sliding up and down the rod as the execution of the movement lengthens or shortens the range of the arm. In the case of a paralysed arm the nurse or orderly supervising the treatment puts the machine in motion.

Fig. 1.

### **Arm Flexion and Extension.**—Fig. 2.

The essential part of this machine is the arm lever, which, by means of a weight sliding along the graduated lever, can be so adjusted as to give any desired resistance from zero to maximum. In order further to meet the requirements and range of movement of each particular case, the seat can also be adjusted. The machine is designed both for active and passive movements.

Fig. 2.

**Finger Flexion and Extension.**—FIG. 3.

Each finger is placed separately into a grip, and by rotation of the lever the natural movements

FIG. 3.

of the finger joints are carried out; the pressure can be adjusted as in the other machines.

**Hip Flexion and Extension.**—FIG. 4.

The patient is seated on an adjustable chair, and the leg is fitted into the half-circle supports shown in the illustration. By means of a weight sliding along the graduated lever the machine can be so

FIG. 4.

adjusted as to allow of passive and active movements of the hip joint. The patient executes an active movement of flexion of the hip, and the weight, displaced from its position of equilibrium, will bring down the leg into the vertical position, and bringing with it the hip will make the patient execute a passive movement the inverse of the active movement.

**Knee Flexion and Extension.**—FIG. 5.

The patient is comfortably seated, and his lower limbs are placed in fittings provided for the purpose,

FIG. 5.

and the weights are adjusted to the desired movements either for active or passive movements.

**Foot Circumduction.**—FIG. 6.

The patient is seated in front of the machine, and the foot is fitted into the "foot shape" shown in the diagram at the left. Rotatory movements in the ankle joints are carried out either actively by the patient's own efforts, or passively by turning

FIG. 6.

FIG. 7.

the handle shown in the illustration. The same system of adjustment by means of a sliding weight is also adapted to this machine. This appliance is used in cases of stiffness of the ankle joint and in paralysis or lameness of the muscles of the foot.

**Pronation and Supination of Forearm.**—FIG. 7.

The patient stands in front of the machine, and, taking hold of the handle shown in the illustration, performs rotatory movements.

The Zander machines are manufactured by Messrs. Spencer, Heath, and George, Goswell Road, London.

## Figures and Tables

**Fig. 1. f1:**
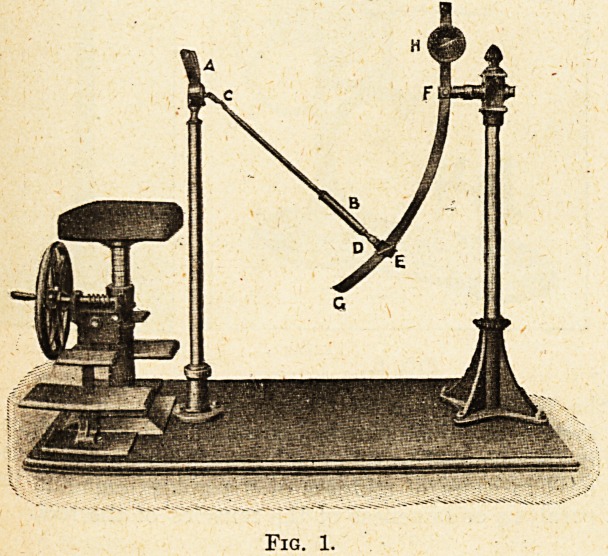


**Fig. 2. f2:**
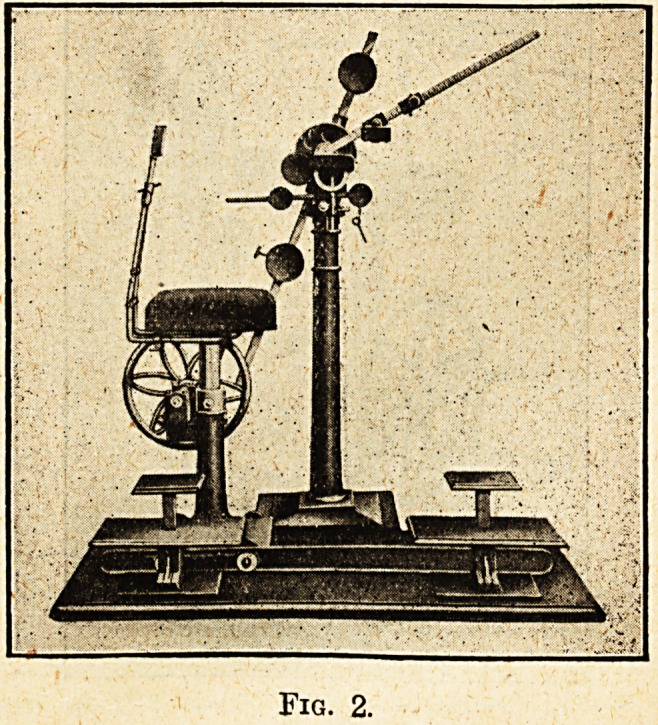


**Fig. 3. f3:**
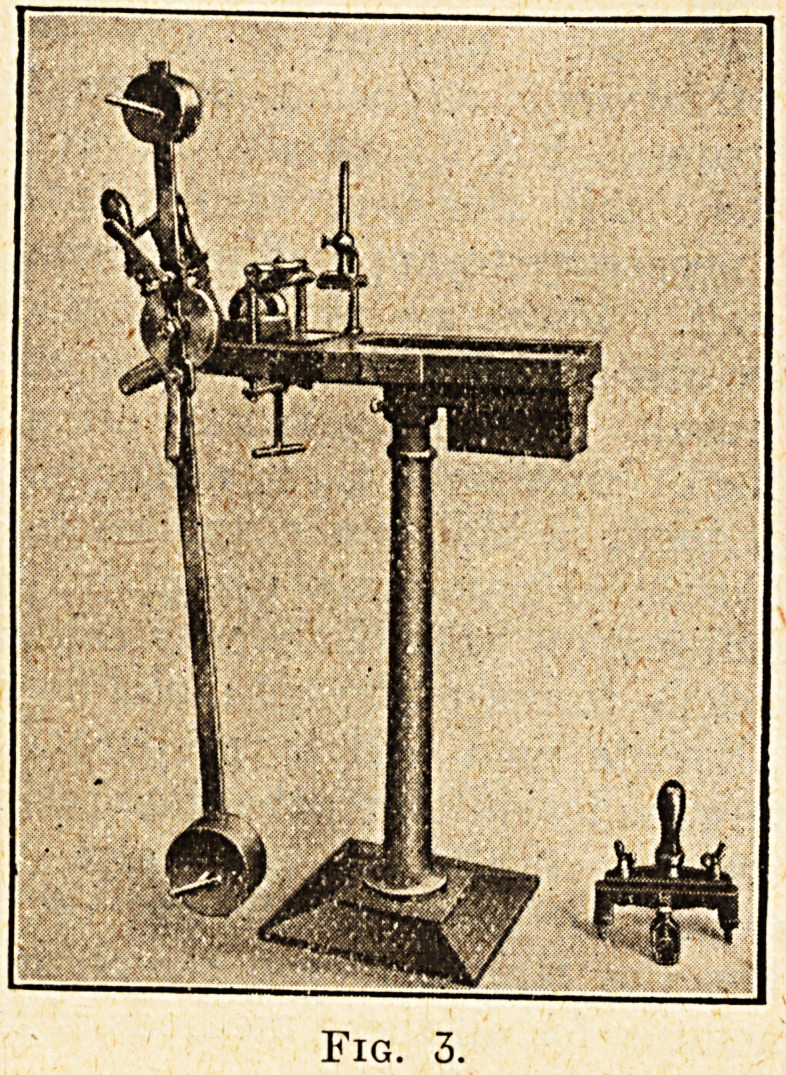


**Fig. 4. f4:**
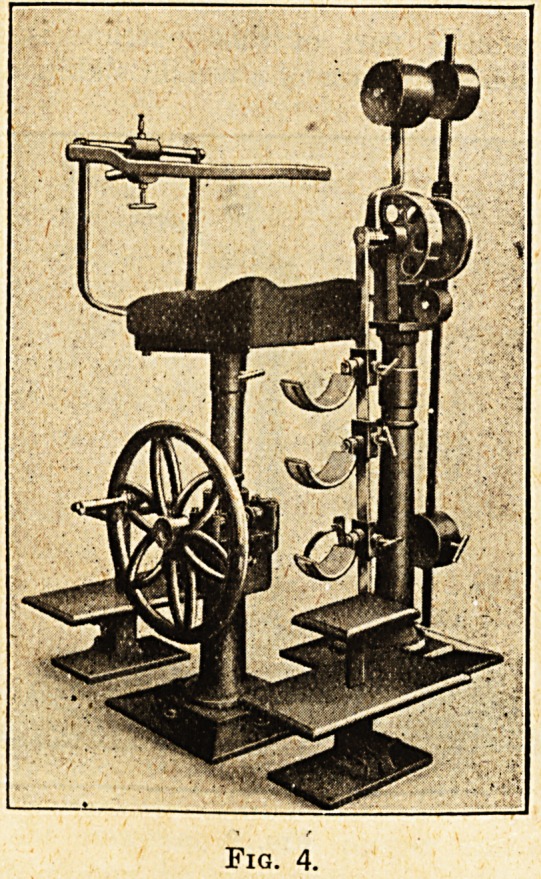


**Fig. 5. f5:**
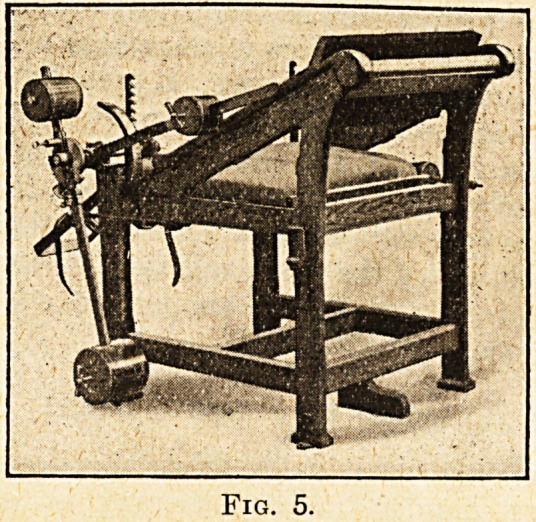


**Fig. 6. f6:**
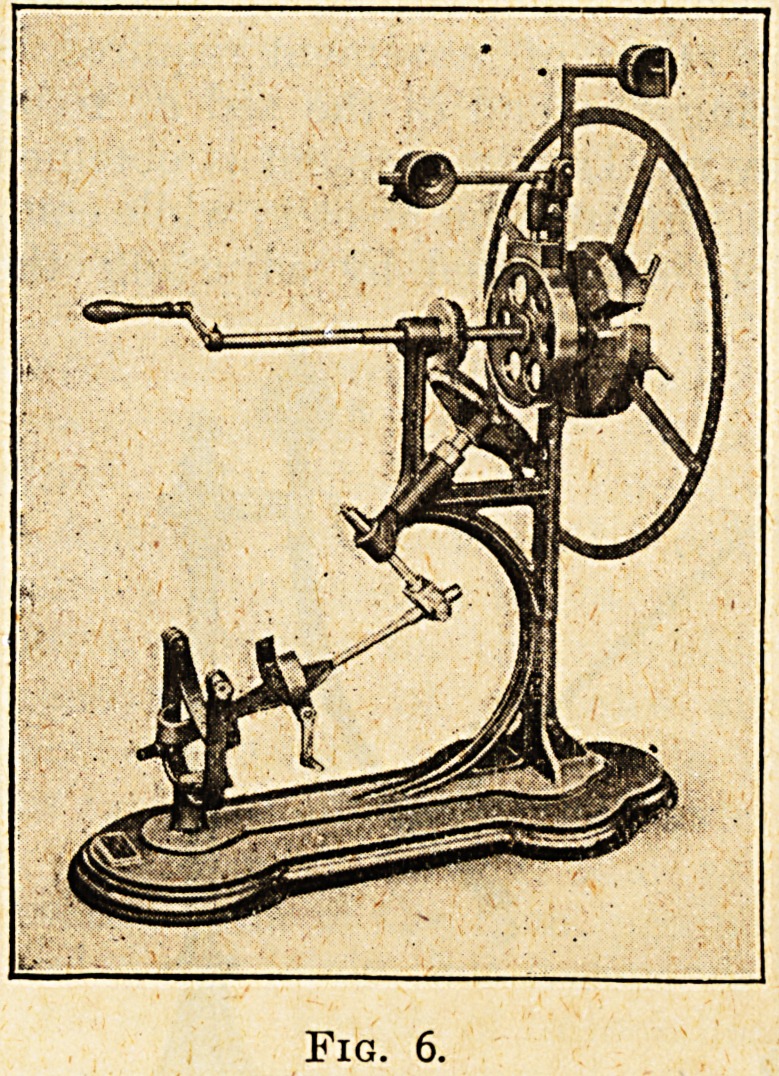


**Fig. 7. f7:**